# Rethinking PbtO₂ responses to hyperoxemia: laying the groundwork for a new approach to multimodal neuromonitoring

**DOI:** 10.1186/s13054-025-05502-8

**Published:** 2025-10-09

**Authors:** Gurgen Harutyunyan Hovhanisyan, Garnik Harutyunyan Jaghatspanyan, Suren Soghomonyan

**Affiliations:** 1 Hospital 9 de Octubre, VITHAS, Emergency Department, Valle de la Ballestera 59, Valencia, 46015 Spain; 2 Faculty of Pharmacy, Universitat De València, Valencia, Spain; 3 Clinical Department of Anesthesiology, The Ohio State University Wexner Medical Center, Columbus, Ohio USA

## Abstract

The interpretation of brain tissue oxygen tension (PbtO₂) in neurocritical care remains controversial, particularly during hyperoxemic conditions. In this comment on the article by Bögli et al., we propose that the observed rise in PbtO₂ following increased FiO₂ may be better explained by the conformational transition of hemoglobin from the relaxed (R) to the tense (T) state at the end of cerebral capillaries. This shift, which enhances oxygen release and buffering capacity, helps maintain arterial-like oxygen tension despite low venous oxygen saturation. We discuss the implications of this mechanism for understanding multimodal neuromonitoring (MMM) data, the effects of cerebral autoregulation, and the role of blood storage lesions. Recognizing hemoglobin conformation as a physiological determinant may help refine MMM thresholds and neuroprotective strategies in traumatic brain injury.

In their article on multimodal monitoring for traumatic brain injury management, Stefan Yu Bögli and colleagues state that brain tissue oxygen tension (PbtO₂) increases as a result of elevated levels of dissolved arterial oxygen when the fraction of inspired oxygen (FiO₂) is increased: “since hemoglobin is often already saturated, but additional oxygen is dissolved within the blood” [1]. However, the mechanisms underlying the distinct increase in PbtO₂ following FiO₂ elevation remain debated. According to Johnston and colleagues, PbtO₂ reflects end-capillary oxygen tension [2], which aligns with physical principles such as the equilibration of partial pressures in connected compartments. In contrast, Gargadennec and colleagues propose an alternative explanation: “an increase in interstitial oxygen diffusion at the arterial capillary side” [3].

What may initially appear as a minor artifact could significantly affect the interpretation of multimodal monitoring (MMM) data. First, the amount of dissolved oxygen in arterial blood that increases with higher FiO₂ is minimal and begins to decline at the entrance of the capillary as hemoglobin desaturates. By the end of the capillary, the blood becomes venous due to an oxygen extraction fraction of 30–40%, yet PbtO₂ may still reach around 100 mmHg under hyperoxemic conditions [3], maintaining arterial-like PO₂ values at the capillary end. The coexistence of low venous oxygen saturation (SO₂) with high pericapillary oxygen tension (i.e., PbtO₂) under hyperoxemia can be explained by the conformational transition of hemoglobin from the relaxed (R) to the tense (T) state at the end of cerebral capillaries. The T-state has markedly reduced oxygen affinity and significantly greater buffering capacity [4]. Its shallow, hyperbolic dissociation curve promotes a more uniform oxygen distribution along the capillary, despite the drop in SO₂, thereby preventing the formation of “hypoxic corners” at the distal capillary end—contrary to the predictions of the classical Krogh model.

Second, the increased buffering capacity of hemoglobin in the T-state is essential in human cerebral metabolism, given a respiratory quotient of 1: for each mole of O₂ consumed, one mole of CO₂ is produced, generating nearly one mole of hydrogen ions (H⁺) within erythrocytes, which are buffered exclusively by hemoglobin. However, human hemoglobin cannot buffer more than 0.6 moles of H⁺ per mole of oxygen released—according to the Haldane coefficient. Achieving this maximal buffering capacity is only possible in the T-state [4]. Therefore, in general, the predominance of T-state hemoglobin at the end of cerebral capillaries is likely crucial for maintaining acid–base homeostasis and ensuring homogeneous pericapillary oxygen distribution.

This concept of R-to-T state transition in cerebral capillaries not only explains the high PbtO₂ values observed during hyperoxemia, but also has important implications for the interpretation of MMM data. Below the lower limit of autoregulation, hemoglobin’s buffering capacity becomes depleted due to overloading with CO₂ and CO₂-derived H⁺, triggering a metabolic crisis [5]. Elevated CO₂ levels and the resulting intracellular acidosis directly or indirectly suppress mitochondrial respiration. Above the upper limit, the R-to-T transition of hemoglobin is hindered, leading to hyperperfusion and a progressive loss of blood buffering capacity.

As mentioned by the authors, PbtO₂ values rise sharply while rSO₂ changes remain minimal—suggesting a flat, low-positioned dissociation curve, characteristic of the T-state of hemoglobin. This observation underscores the potential value of daily monitoring of the PbtO₂/rSO₂ relationship and highlights the need for cautious bedside interpretation of PbtO₂ values in neurotrauma patients exposed to minimal hyperoxemia.

The unpredictable changes in PbtO₂ following blood transfusion can also be explained by this model: hemoglobin stored for more than two weeks lacks sufficient 2,3-DPG [6] to stabilize the T-state, leading to reduced PbtO₂ and the formation of “hypoxic corners.” In contrast, transfused blood with high 2,3-DPG levels enhances both PbtO₂ and the buffering capacity of blood, thereby supporting improved metabolic function.

Finally, it is important to recognize CO₂-related vasoreactivity as an integral component of cerebral autoregulation. Hypoventilation impairs hemoglobin buffering capacity through excessive binding of CO₂ and CO₂-derived hydrogen ions—an effect partially counteracted by cerebral vasodilation. In contrast, hyperventilation-induced vasoconstriction may facilitate T-state stabilization at the capillary end by maintaining an adequate level of allosteric effectors.

The distinct increase in measured PbtO₂ during hyperoxemia is likely a consequence of the predominance of tense-state hemoglobin at the end of cerebral capillaries. Preserving the buffering capacity of cerebral blood flow may represent a relevant physiological target for the refinement of multimodal neuromonitoring strategies.

## Data Availability

No datasets were generated or analysed during the current study.
